# Book Notices

**Published:** 1891-11

**Authors:** 


### Book Notices.

A NATIONAL FAMILY PAPER.—The announcements of *The Youth's Companion* for 1892, which we have received, seem to touch about all healthy tastes. Its fiction embraces folklore, serial, sea, adventure and holiday stories. Frank Stockton, Clark Russell, Will Allen Dromgoole, Mary Catherine Lee are a few of the distinguished story-writers.

Its general articles cover a wide range. Self-Education, Business Success, College Success, Girls Who Think they Can Write, Natural History, Railway Life, Boys and Girls at the

World's Fair, Glimpses of Royalty, How to See Great Cities, Practical Advice, are some of the lines to be written on by eminent specialists.

Gladstone, De Lesseps, Vasili Verestchagin, Cyrus W. Field, Andrew Carnegie, Mrs. Henry M. Stanley are among the contributors. *The Companion* readers thus come into personal touch with the people whose greatness makes our age famous. Its 500,000 subscribers show how it is appreciated.

Whoever subscribes now for 1892 gets it free from the time the subscription is received till Jan. 1, 1893. $1.75 a year. Address, *The Youth's Companion*, Boston, Mass.

---

INTERNATIONAL CLINICS.—A quarterly of clinical lectures on *Medicine*, *Surgery*, *Gynecology*, *Pediatrics*, *Neurology*, *Dermatology*, *Laryngology*, *Ophthalmology*, *and Otology*, by professors and lecturers in the leading medical colleges of the United States, Great Britain and Canada. Edited by J. M. Keating, M. D., Philadelphia; J. P. Crozer Griffith, M. D., Philadelphia; J. Mitchell Bruce, M. D., F. R. C. P., London, England; David W. Finlay, M. D., F. R. C. P., London, England. April 1, 1891. Philadelphia: J. B. Lippincott Company.

This book is bound in cloth, well printed and edited, and contains 350 pages. It is is intended to answer for a complete course of post-graduate instructions. Many physicians have the inclination, but not the time, nor means, to take a post-graduate course of lectures. This does not supply that want, but the periodical has in it much merit, and the enterprising editors deserve every success. The contents of volume 1 show thirty-six clinical lectures, on subjects of greatest interest to the practitioner. There are fourteen plates and eighteen figures to illustrate the text. B.

---

SEXUAL NEURASTHENIA.—Its hygiene, causes, symptoms and treatment, with a chapter on diet for the nervous, by Geo. M. Beard, A. M., M. D. (Posthumous manuscript.) Edited by A. D. Rockwell, A. M., M. D., N. Y. E. B. Treat, publisher. Pp. 300. Price $2.75. 1891.

One is disappointed on reading this otherwise very good little book, at not finding more pathology. The work is a little on the sensational order.

---

THE ADVERTISER'S HANDY GUIDE, 1891. Compiled and published by J. H. Bates, Newspaper Advertising Agent, 38 Park Row, New York. Price, $2.

THE JOURNAL has received a copy of Redmondino on Circumcision, and Bœnning's Practical Anatomy, from the publishing house of F. A. Davis, Philadelphia. Both handsome and creditable editions in cloth and gold.

---

THE MODERN ANTIPYRETICS: THEIR ACTION IN HEALTH AND DISEASE. By Isaac Ott, M. D., Johns-Hopkins University. E. D. Vogel, Publisher, Easton, Pa. 1891. pp. 52.

The subject is discussed under the following heads: Fever; chemistry; physiological and pathological action; therapeutics, and value of antithermics in typhoid fever. B.

---

DIABETES: ITS CAUSES, SYMPTOMS AND TREATMENT. By Chas. W. Purdy, M. D., Queen's University; author of "Bright's Disease and Allied Affections of the Kidneys," etc. With clinical illustrations. F. A. Davis, Philadelphia and London, publisher. pp. 175. Price, $1.

This book is No. 8 in the Physicians' and Students' Ready Reference Series, gotten out by the above enterprising publisher. It is a well prepared article on the diabetes, and will repay a close reading. In thirty States and Territories of the United States, during the year 1880, there were 1,443 deaths from this disease, or 1.90 per 1000 deaths. Maine and Vermont give the greatest death rate; 4.41 and 6.36 per 1000 deaths respectively. The author claims that the greatest death rate is found in the higher altitudes, with low thermometric range. Nothing has been added to the pathology and treatment, yet the work is a good review of the subject. About a third of the book is devoted to clinical considerations and to diabetes insipidus. B.

---

THE INTERNATIONAL MEDICAL JOURNAL AND PRACTITIONER'S INDEX; a Work of Reference for Medical Practitioners. Ninth year. 1891. New York: E. B. Treat, publisher. Pp. 580. Price, $2.75. Edited by P. W. Williams, M. D., Secretary of Staff, assisted by a corps of thirty-eight collaborators,—European and American,—specialists in their several departments.

The peculiarity of this valuable "Annual" is that every year excels the preceding in the contents and mechanism. It presents a valuable and handy resume of medical progress as gleaned from the journal articles of the year.

The book is divided into four parts. I., is devoted to New Remedies and Therapeutics; II., to Diagnosis; III., New Treatment and Retrospect; IV., Recent Improvements in Sanitation, Pharmacy, books of the year, etc. The whole book is alphabetically arranged and is well indexed. The last part of the book gives a valuable resume of the results of treatment af alcoholic inebriates in asylums. B.

